# The role of miR-320 in glucose and lipid metabolism disorder-associated diseases

**DOI:** 10.7150/ijbs.53419

**Published:** 2021-01-01

**Authors:** Hengzhi Du, Yanru Zhao, Zhongwei Yin, Dao Wen Wang, Chen Chen

**Affiliations:** Division of Cardiology, Hubei Key Laboratory of Genetics and Molecular Mechanisms of Cardiological Disorders, Tongji Hospital, Tongji Medical College, Huazhong University of Science and Technology, Wuhan 430030, China

**Keywords:** miRNA, glucose and lipid metabolism, diseases

## Abstract

Glucose and lipids are important nutrients that provide the majority of energy for each organ to maintain homeostasis of the body. With the continuous improvement in living standards, the incidence of metabolic disorder-associated diseases, such as diabetes, hyperlipidemia, and atherosclerosis, is increasing worldwide. Among them, diabetes, which could be induced by both glucose and lipid metabolic disorders, is one of the five diseases with the highest incidence and mortality worldwide. However, the detailed molecular mechanisms underlying glucose and lipid metabolism disorders and target-organ damage are still not fully defined. MicroRNAs (miRNAs) are small, non-coding, single-stranded RNAs, which usually affect their target mRNAs in the cytoplasm by post-transcriptional regulation. Previously, we have found that miR-320 contributed to glucose and lipid metabolism via different signaling pathways. Most importantly, we identified that nuclear miR-320 mediated diabetes-induced cardiac dysfunction by activating the transcription of fatty acid metabolic genes to cause lipotoxicity in the heart. Here, we reviewed the roles of miR-320 in glucose and lipid metabolism and target-organ damage.

## Introduction

Glucose metabolism and lipid metabolism usually interact with each other. Glucose and lipid metabolism stability is important for maintaining the function of all organs of the body. However, with the rapid increase in the prevalence of high-fat or high-glucose diets, glucose and lipid metabolism-associated diseases have attracted increasing attention 1. For example, glucose and lipid metabolism disorder is involved in various diseases such as obesity 2, diabetes mellitus 3, hypertension 4, dyslipidemia 5, nonalcoholic fatty liver disease 6, and atherosclerosis 7. Glucose and lipid metabolism-associated diseases also participate in the occurrence of their complications. For example, extreme hyperglycemia may lead to ketoacidosis, which damages the vasculature and nervous system 8. Moreover, the glucose and lipid metabolism disorder in diabetes can destroy various organs, especially the eyes, kidneys, nerves, heart and blood vessels 9. However, the underlying molecular mechanisms of glucose and lipid metabolism and its involvement in multiple target-organ damage are not fully explored.

MicroRNAs (miRNAs) are small conserved non-coding RNAs of 19-25 nucleotides that have crucial effects on gene regulation in various biological processes and diseases mainly by inhibiting target mRNA translation or promoting target mRNA degradation 10. Initial miRNA-associated studies were mainly focused on cancer development, cardiovascular diseases, and metabolic diseases. It was found that the expression profile of miRNAs was changed in plasma and tissue samples under disease conditions such as cancer 11, hyperlipidemia 12, hyperglycemia 13, endothelial cell dysfunction 14 and diabetes 15. We also identified the altered expression of circulating miRNAs in patients with coronary heart disease (CHD), compared with healthy individuals, among which the expression level of miR-320 was positively correlated with the risk of CHD 16. The functions of these changed miRNAs including miR-320 were gradually discovered in various diseases. For example, nuclear miR-320 mediated diabetes-induced cardiac dysfunction by activating transcription of fatty acid metabolic genes to cause lipotoxicity in the heart 17.

In humans, the miR-320 family consists of miR-320a (chr8), miR-320b (chr1), miR-320c (chr18), miR-320d (chr13 and chrX), and 320e (chr19), among which miR-320a is present in all primates 18. miR-320 is encoded directly upstream of the cell cycle gene POLR3D, which is a conserved subunit specific to RNA polymerase III 19, 20.

Among these metabolism associated-miRNAs, our previous study showed that miR-320 was markedly elevated in the peripheral blood of patients with CHD 16. Meanwhile, we discovered various roles of miR-320 in metabolism-associated injuries including aggravating diabetic cardiac dysfunction 17, promoting atherosclerosis 16 and deteriorating diabetic nephropathy 21. In this review, we focused on the current knowledge about the roles of miR-320 in glucose and lipid metabolism and the target-organ damage.

## miR-320 serves as a potential biomarker for glucose and lipid metabolism-associated diseases

It has been clarified that miRNAs are highly stable in bodily fluids, which makes them feasible candidates for non-invasive biomarkers 22. The expression profiles of miRNAs in clinical specimens could be identified by high-throughput RNA sequencing or real-time PCR assays with high sensitivity and specificity 23. Accumulating evidence suggests that miRNAs can be used as potential biomarkers for disease diagnosis and prognosis 24, 25. Regarding the metabolism of glucose and lipid, multiple studies have found that the expression level of miR-320 was changed in the blood or tissues from patients or animal disease models with metabolic syndrome 26, diabetes and its complications 27.

### miR-320 is a potential biomarker for diabetes

Diabetes is a systemic metabolic disease characterized by insulin resistance and relative underproduction of insulin 28, which in turn will impair the function of other organs including the heart, liver and kidney through systemic metabolic disorders 29, such as hyperglycemia and dyslipidemia 30, 31. The development of biomarkers for early diagnosis could be helpful in identifying individuals at risk of developing diabetes or related complications 27. The traditional diagnosis of type 2 diabetes (T2DM) is generally based on the biochemical examinations of fasting plasma glucose (FPG) 32, oral glucose tolerance test (OGTT) and glycated hemoglobin (HbA1C) levels 33. Moreover, C-reactive protein, adipokines, and incretins were regarded as candidate novel biomarkers for T2DM 34, 35. However, the risk assessment of related complications could not be defined by these biomarkers, which requires the discovery of novel biomarkers 36.

To explore the potential molecules that could predict the development and progression of diabetes mellitus, multiple studies have analyzed the miRNA profiles in serum, plasma or blood cells to find candidate miRNAs 37, 38. In 2010, Zampetaki et al. identified the T2DM-related expression pattern of circulating miRNAs by analyzing blood samples from over 800 individuals randomly selected from the Bruneck population (Bolzano Province, Italy) for the first time 37. This prospective study revealed lower plasma levels of miR-320 in 80 individuals with either pre-diabetes or T2DM. It was noteworthy that the change in miR-320 was earlier than the diagnosis of T2DM, which suggested a possibility that circulating miR-320 might be a predictor for the ongoing T2DM and its complications. On the other hand, Karolina et al. detected an upregulation of circulating miR-320 in patients with T2DM and observed a positive correlation between the miR-320 level and fasting blood glucose level by measuring miRNA expression in the blood and exosomes from 265 patients with different health conditions associated with metabolic syndrome 26. In addition to the expression profile of miRNAs in diabetes, Ling et al. have shown that miR-320 was also upregulated in insulin-resistant 3T3-L1 adipocytes compared with normal adipocytes 39. It is worth mentioning that Zampetaki et al. detected a downregulation of circulating miR-320 in patients with T2DM, which is contradictory to the findings of others. The reasons for these different observations might be the following: 1) the detection method and 2) the limited sample size or the different cohorts enrolled in various studies. Specifically, instead of testing each sample individually, Zampetaki et al. pooled the samples to detect miRNA levels in plasma; this sampling strategy might lead to the overall decrease because of certain particular extremum. The limited sample size or the different cohorts may also have contributed to the differences observed. For example, Zampetaki et al. identified multiple candidate miRNAs, including decreased plasma miR-20b and miR-150, in 80 prevalent DM individuals. However, others also reported increased circulating exosomal miR-20b and miR-150 (n = 16) 40, as well as increased plasma miR-150 (n = 462) in patients with CDH who developed DM 27. Therefore, it is necessary to further expand the sample size and strictly design prospective studies to identify whether miR-320 could serve as a biomarker for diabetes.

### miR-320 is a potential biomarker for atherosclerosis

Atherosclerosis is a chronic disease characterized by the accumulation of plaques, which are characterized by large necrotic cores with thin fibrous caps, cholesterol deposits, inflammatory cells and calcifications within the arteries 41, and is a leading cause of death and loss of productive life years worldwide 42. Currently, imaging biomarkers are the major method to diagnose atherosclerosis clinically 43. The traditional way to diagnose atherosclerosis is by using ultrasound, which may be subjective. In recent years, computed tomography angiography (CTA) has also been commonly used, but there are still defects such as contrast agent allergy, false positives, and limited assessment of the plaque rupture or vascular blockage risk. Some studies observed that the miRNA expression profiles were changed in patients with coronary atherosclerosis, which might be potential biomarkers for the diagnosis and prognosis of atherosclerosis 44, 45. For example, Wang et al. detected 9 candidate miRNAs in the plasma exosome from 42 patients with coronary atherosclerosis and identified miR-30e as a novel diagnostic biomarker for coronary atherosclerosis 46.

Coronary heart disease (CHD), mainly derived from the progression of atherosclerosis, is also one of the leading causes of death worldwide 47, 48. It was reported that individuals with diabetes mellitus suffer from a higher incidence of CHD compared with those without diabetes 49. Furthermore, the major cause of death in patients with CHD is the rupture of atherosclerotic plaques leading to the formation of a thrombus and causing a myocardial infarct. However, the current diagnosis of CHD is established by coronary angiography or CTA, which have shown limited information for the early diagnosis and prognosis assessment of CHD 50, 51.

With the study on CHD, researchers found that miRNAs could be potential biomarkers for the early diagnosis and prognosis assessment of CHD. For example, Zhou et al. performed miRNA microarray analysis in the plasma of 3 patients with CHD patients and 3 healthy controls and observed that miR-206 and miR-574-5p were significantly upregulated in patients with CHD by performing quantitative real-time PCR (qRT-PCR) analysis of miRNA expression in plasma of another 67 patients with CAD and 67 healthy controls, which suggested the sensitive and specific diagnostic value of miRNAs for CHD 52. Zhong et al. identified that miR-142-3p and miR-17-5p might be potential targets for follow-up research in evaluating biomarkers of CHD by analyzing the miRNA expression profiles of circulating miRNAs from 12 patients with CHD and 6 healthy controls 53. However, it is worth mentioning that the sample number adopted in the above studies is limited and it would be better to increase the number of specimens, thereby increasing the confidence, which would make the results of the studies more convincing. Meanwhile, the expression of miR-320 was also changed in the patients with CHD. In a previous study from our group, we performed miRNA microarray analysis in the peripheral blood of 10 patients with CHD, 10 CHD high-risk individuals and 10 healthy controls and found that miR-320 was highly expressed in patients with CHD 16. Moreover, miR-320 was a key regulator contributing to multiple aspects of atherogenesis, which suggested that miR-320 might be a potential biomarker for atherogenesis and CHD 16. Mechanically, we found that SP1 could transcriptionally up-regulate hsa-miR-320a expression in endothelium cell 16. Recently, Su et al. profiled miRNAs in the plasma from 203 patients with CHD and 144 age-matched controls (126 high-risk controls and 18 healthy volunteers) and showed that miR-320e was increased in CHD patients 54. Moreover, correlation analysis indicated that miR-320e could be a better biomarker for CHD diagnosis compared to most conventional clinical factors, such as apolipoprotein A (ApoA), apolipoprotein B (ApoB), and LPA 54. Meanwhile, Liu et al. also performed miRNA microarray analysis in an OPG^-/-^ mouse model to identify miRNAs involved in vascular calcification, a major characteristic of atherosclerosis 55. They found that miR-320 was decreased compared with control mice and suggested that miR-320 might play an important role in the progression of vascular calcification 55.

It is worth mentioning that hyperlipidemia, a group of disorders characterized by excessive lipids, is an independent risk factor for diabetes and CHD 56. Many studies have shown that patients with diabetes or CHD have lipid metabolic disorders including elevated plasma total cholesterol, triacylglycerol (TRG), and LDL and decreased HDL 57, 58. Interestingly, researchers have found that miR-320 might be a highly specific miRNA for risk assessment of hyperlipidemia. For example, Xu et al. collected the serum samples and clinical data of 122 patients with hyperlipidemia and 168 healthy subjects to detect the circulating miRNA profile by miRNA microarray analysis 12. They detected 22 changed miRNAs, including upregulation of miR-320b, miR-320e, miRNA-191-3p, miRNA-933, and miRNA-425-3p, in the peripheral circulation of patients with lipid metabolism disorder, and these miRNAs could be risk assessment factors for hyperlipidemia.

### miR-320 is a potential biomarker for adiposity

With the rapid increase in the prevalence of high-fat or high-glucose diets, adiposity, which often induces insulin resistance, type 2 diabetes, and cardiovascular diseases, has become a major public health problem worldwide 59. To investigate whether miRNAs are involved in adiposity, researchers performed miRNA expression analysis in human samples 60-62. For example, Pescador et al. collected the serum samples and clinical data of 30 patients with type 2 diabetes, 20 obese patients, 16 obese patients with type 2 diabetes and 20 healthy controls to detect the changed circulating miRNAs by miRNA microarray analysis 63. They discovered that 3 microRNAs, namely, miR-138, miR-15b, and miR-376a, were potential predictive biomarkers in obesity. Meanwhile, Eiji et al. also observed that miR-320 might be a biomarker for obesity 64. They analyzed the circulating miRNAs profile in 526 individuals who participated in health examinations and found that the altered expression levels of miR-320 were significantly associated with visceral adipose tissue levels, which suggested that miR-320 might be a potential biomarker for adiposity.

### miR-320 is a potential biomarker for nonalcoholic fatty liver disease

**Figure 1:**
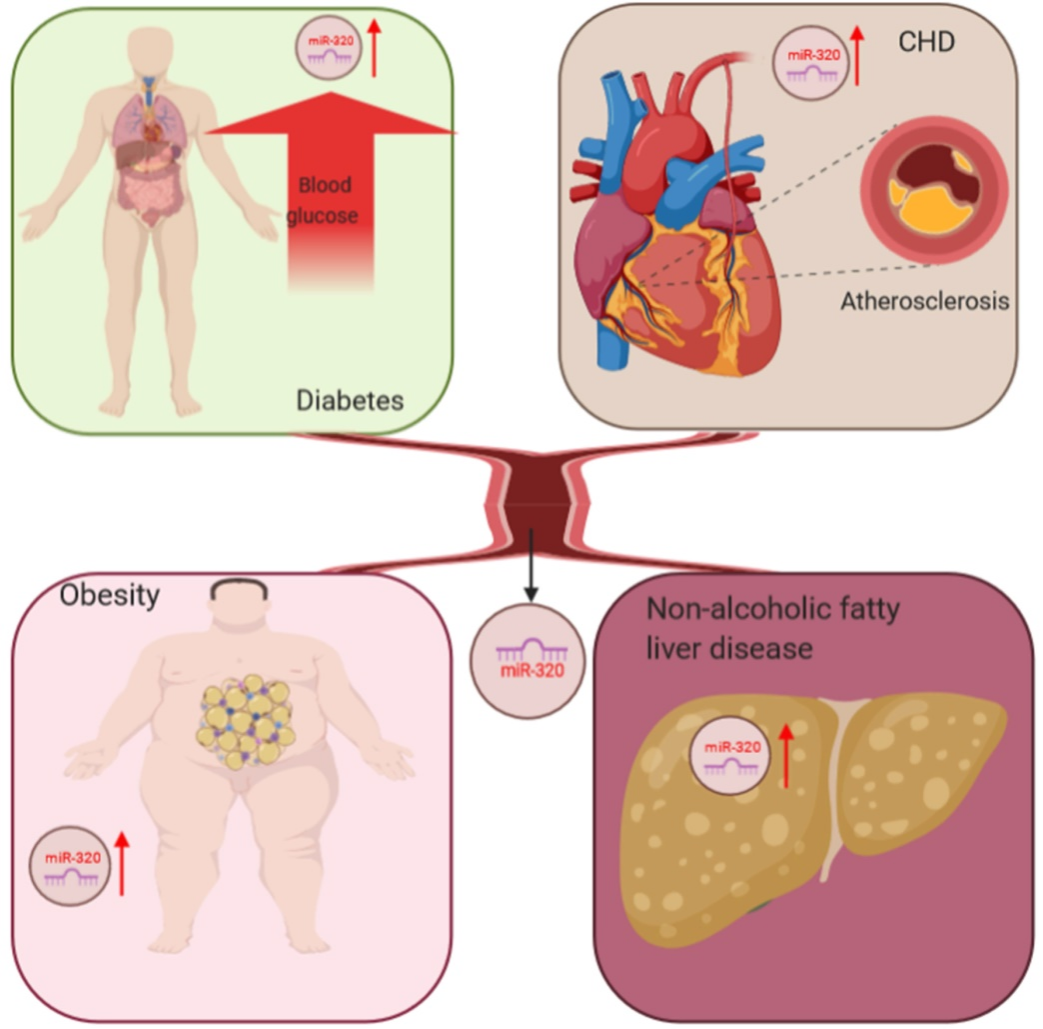
miR-320 serves as a potential biomarker for glucose and lipid metabolism disorder-associated diseases, including adiposity and atherosclerosis, coronary heart disease and nonalcoholic fatty liver disease.

The liver is the most important organ in the body to maintain lipid homeostasis, and hyperlipidemia can further aggravate the accumulation of lipids in hepatocytes leading to nonalcoholic fatty liver disease 65. Kagawa et al. collected plasma samples from steatosis model mice and found upregulated miR-320 by RNA-sequencing, which suggested that miR-320 might be a potential biomarker for nonalcoholic fatty liver disease 66. In conclusion, the abnormal expression of miR-320 was detected in many metabolic diseases, including diabetes and its complications, through miRNA microarray analysis, which indicated that miR-320 might be a potential biomarker for these diseases (Figure 1).

## The effects of miR-320 on glucose and lipid metabolism-associated diseases

By analyzing the miRNA expression profile in diseases related to glucose and lipid metabolic disorders, researchers have found that miR-320 might be a potential biomarker. Further, the biological functions of miR-320 under these conditions were investigated.

### The effects of miR-320 on glucose and lipid metabolism in different target cells

Different cell types may contribute to certain pathological processes via multiple mechanisms. Here, we summarized the effects of miR-320 on glucose and lipid metabolism in white adipocytes, cardiomyocytes, endothelial cells and cancer cells.

#### The effects of miR-320 on glucose and lipid metabolism in white adipocytes

Adipocytes in the body have a variety of important physiological functions including storing triglycerides, maintaining blood glucose homeostasis, and providing energy as the body needs it. These processes are regulated by insulin directly 67. Under normal conditions, insulin binds to the insulin receptor on the plasma membrane of adipocytes 68 and initiates downstream metabolic signaling by recruiting phosphotyrosine-binding scaffold proteins, which in turn activate phosphoinositide-3-kinase (PI3K) 69-72. The critical physiological functions of this signaling pathway in white adipose tissue include the suppression of lipolysis and stimulation of glucose uptake and lipogenesis 67. It was reported that miR-320 could suppress this signaling pathway by targeting the p85 subunit of PI3K 39. Using miRNA microarray analysis, Ling et al. compared miRNA expression profiles between normal insulin-sensitive 3T3-L1 adipocytes and 3T3-L1 adipocytes rendered insulin resistant following treatment with high glucose and high insulin and found that the expression of miR-320 increased significantly compared with the control group. In addition, they found that the p85 subunit of PI3K was a potential target of miR-320 in adipocytes, which suggested that miR-320 could suppress the insulin-PI3K pathway to affect glucose and lipid metabolism.

In addition to the abovementioned approaches, miR-320 can also affect the glucose and lipid metabolism of adipocytes through the endoplasmic reticulum (ER) stress pathway 73. The ER is a critical organelle for protein synthesis, folding and modification, lipid synthesis and calcium storage 74. Due to aging, genetic mutation or environmental stress, unfolded proteins accumulate in the ER 75, which will induce ER stress, the disruption of ion balance in the ER or serve as an obstacle to protein processing and transportation 76. It has been reported that ER stress could lead to decreased insulin action by disrupting insulin receptor signaling 77 and induce lipolysis in adipocytes to maintain energy homeostasis 78. Interestingly, Lu et al. indicated that the expression level of miR-320 in omental adipose tissues and blood samples of 24 obese patients was upregulated compared with healthy donors. Further, they showed that inhibition of the upregulated miR-320 could ameliorate ER stress and the inflammatory response in 3T3-L1 adipocytes.

**Figure 2:**
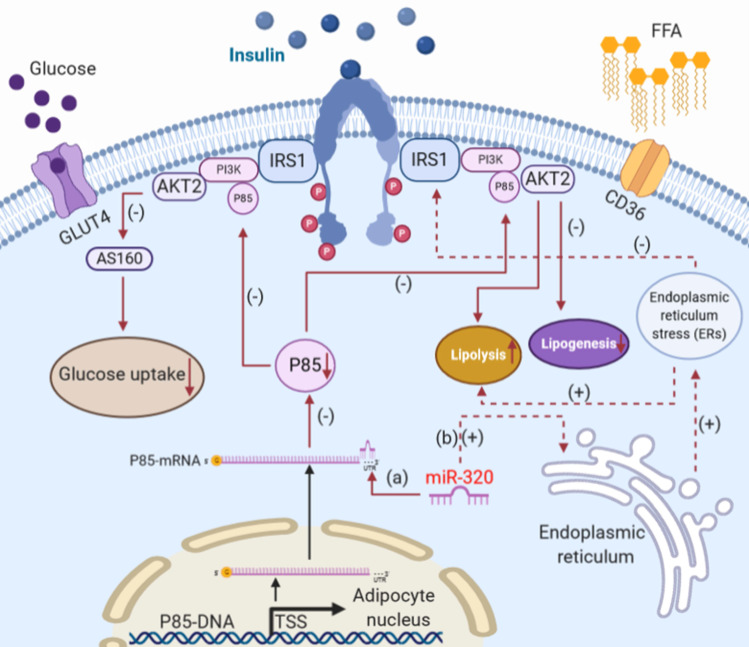
The effects of miR-320 on glucose and lipid metabolism in white adipocytes. The most critical physiological functions of insulin action in white adipose tissue are suppression of lipolysis and stimulation of glucose uptake through translocation of GLUT4 to the plasma membrane. (a) miR-320 could target the p85 subunit of PI3K to induce insulin resistance and suppress glucose uptake and lipogenesis and affect the process of lipolysis. (b) miR-320 induces ER stress to reduce the action of insulin and promote lipolysis. PI3-K, phosphoinositide-3-kinase; GLUT4, glucose transporter 4; IRS1, insulin receptor 1.

Together, the increased expression of miR-320 in stressed adipocytes suppressed glucose uptake and lipogenesis while inducing lipolysis via the insulin-PI3K pathway and ER stress signaling (Figure 2).

#### The effects of miR-320 on glucose and lipid metabolism in cardiomyocytes

Under normal circumstances, the heart needs a continuous energy supply to support both electrical and mechanical activities, which are mainly produced by mitochondrial oxidative phosphorylation 79. The substrate of oxidative phosphorylation is acetyl-CoA, which comes from the oxidation of fatty acids and pyruvate 80, 81. Therefore, mitochondrial dysfunction could affect glucose and lipid metabolism, reducing the normal physiological activities of cardiomyocytes. It has also been reported that miR-320 could induce mitochondrial apoptosis 82. In a mouse model of ischemia-reperfusion injury (I/R), Tian et al. found that the level of miR-320 is substantially higher than that in the corresponding controls and the loss of mitochondrial membrane potential was enhanced. Additionally, they found that AKIP1 was the target of miR-320. AKIP1 plays an essential role in maintaining the function of mitochondria including promoting mitochondrial respiration and improving mitochondrial coupling efficiency while simultaneously attenuating mitochondrial ROS emissions 83-86. The overexpression of miR-320 could inhibit the expression of AKIP1, which might induce the mitochondrial apoptotic pathway and influence glucose and lipid metabolism in cardiomyocytes in the case of ischemia-reperfusion (I/R) injury.

Interestingly, in addition to the pathway described above, miR-320 plays an essential role in cardiomyocyte apoptosis via other signals. Song et al. found that the expression level of miR-320 was significantly upregulated in a rat model of myocardial I/R injury 87. In I/R injury, miR-320 induced cardiomyocyte apoptosis by reducing the expression of IGF-1 88. It is remarkable that insulin significantly reduced the mortality of patients with myocardial ischemia 89, 90. Using an ischemia cell model, Ni et al. found that miR-320 was significantly upregulated after ischemia but decreased to basal levels after insulin pretreatment, suggesting that inhibition of miR-320 was involved in the cardioprotective effects of insulin against myocardial ischemia 91. They also found that the target of miR-320 was Survivin, a key regulator in cell apoptosis 92, 93. Regarding myocardial ischemia, these findings taken together suggest that miR-320 could enhance the apoptosis of cardiomyocytes via multiple pathways.

Although insulin could protect cardiomyocytes as described above, this effect is weakened or absent in diabetes. Moreover, in the early stages of diabetes, fatty acid intake in cardiomyocytes by CD36 is increased due to the lack of insulin or insulin resistance 29, 94. Recently, we found the miR-320 could promote the process of fatty acid intake in cardiomyocytes 17. The upregulation of miR-320 was observed in cardiomyocytes from db/db mice compared to controls. Interestingly, we noted that miR-320 was not only present in the cytoplasm but also in the nucleus of mouse myocytes. Moreover, we revealed that miR-320 could directly target to the CD36 promoter by forming a complex with Ago2 to enhance CD36 transcription, resulting in increased fatty acid transport. To further validate the function of nuclear miR-320 in cardiomyocytes, the Ago2-knockdown cardiomyocyte cell line was established using CRISPR-Cas9 technology. We found that CRISPR-Cas9-mediated Ago2 knockdown abolished miR-320-induced transcriptional remodeling, and nuclear Ago2 re-expression restored the effects of miR-320 in the nucleus 95. In addition, further analysis in our study suggested that Ago2/miR-320 complexes could target Cep57 or Fscn2 promoter DNA for transcriptional regulation and affect the process of glucose and lipid metabolism. It is worth noting that there has been no report on the effect of Cep57 or Fscn2 on glucose and lipid metabolism. Despite the increased FFA use in diabetic hearts, excessive fatty acid leads to intracellular lipid accumulation, which activates PPAR-α signaling, leading to the increased transcription of genes involved in FFA oxidation and the excess generation of reactive oxygen species (ROS) in mitochondria 96-98. Moreover, it has been proven that PPAR-α signaling could increase the expression of CD36 to improve the transport of FFA 99, which might synergize the effects of miR-320 in cardiomyocytes.

**Figure 3:**
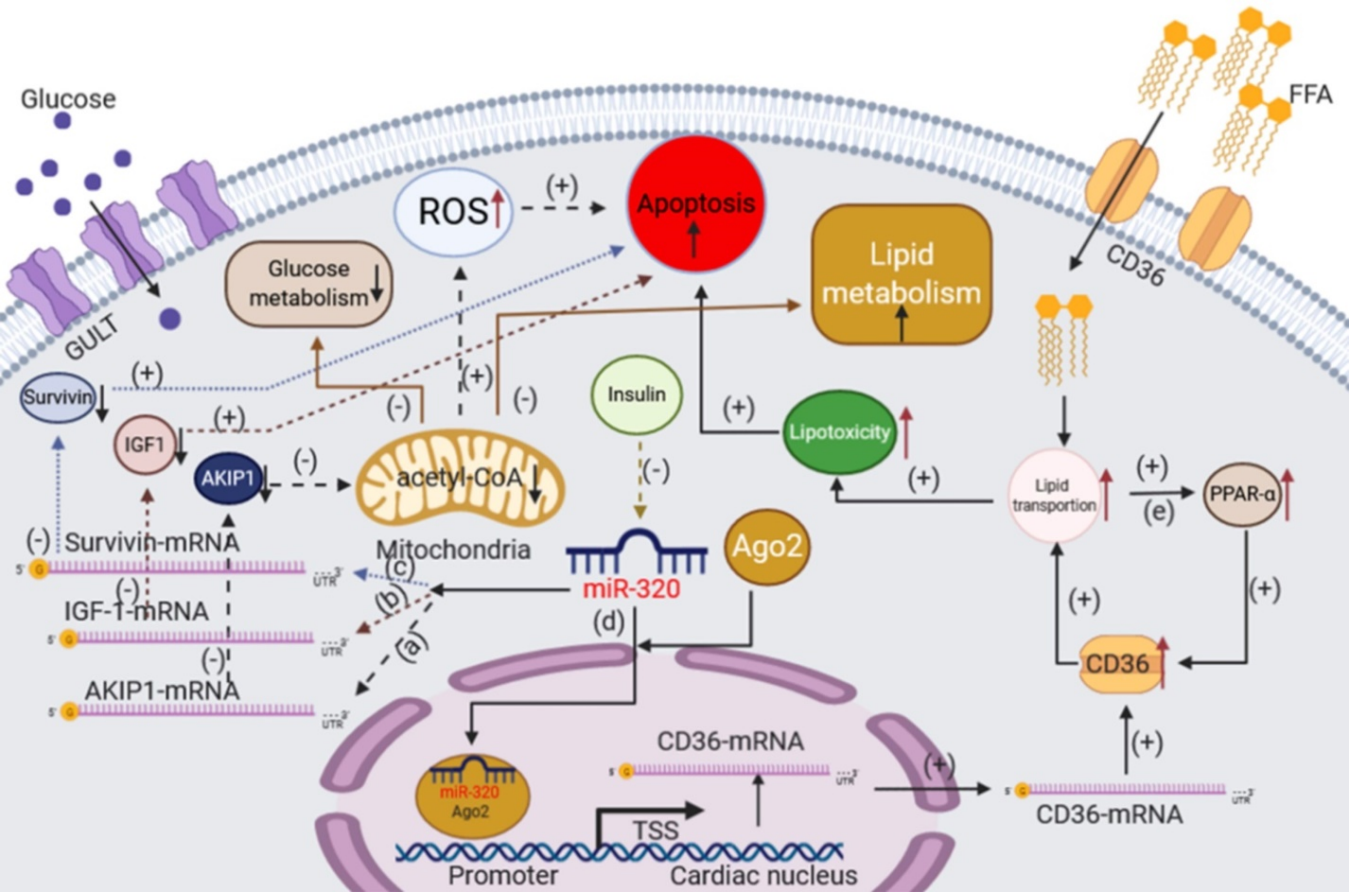
The effects of miR-320 on glucose and lipid metabolism in cardiomyocytes. (a) In an ischemia-reperfusion injury (I/R) model, miR-320 could target AKIP1 to induce the mitochondrial apoptotic pathway and suppress the oxidative metabolism of glucose and lipid in cardiomyocytes. (b) miR-320 reduces the expression of IGF-1 to induce cardiomyocyte apoptosis in the case of I/R injury. (c) miR-320 reduces the expression of Survivin to induce cardiomyocyte apoptosis. (d) miR-320 targets to the CD36 promoter directly by forming a complex with Ago2 to enhance CD36 transcription and promote fatty acid transport. (e) The lipid accumulation activates PPAR-α signaling leading to the increased transcription of many genes involved in FFA oxidation and increases the expression of CD36 to improve the transport of FFA. AKIP1, A kinase interacting protein 1; IGF-1, insulin-like growth factor 1.

In conclusion, miR-320 could damage cardiomyocytes not only by promoting apoptosis but also via glucose and lipid metabolism disorder (Figure 3).

#### The effects of miR-320 on glucose and lipid metabolism in endothelial cells

Damage to myocardial microvascular endothelial cells (MMVECs) is one of the key processes in diabetic cardiomyopathy 100, 101, which leads to insufficient myocardial angiogenesis, represented by reduced coronary artery collateral vessel formation and low capillary density 102, 103. In addition to affecting glucose and lipid metabolism in cardiomyocytes, miR-320 was also functional in endothelial cells in diabetic cardiomyopathy 16.

As mentioned above, we found that miR-320 is a potential biomarker for atherosclerosis and CHD. Moreover, we discovered that miR-320 inhibited proliferation and induced apoptosis of endothelial cells by targeting Serum Response Factor (SRF) 16. Meanwhile, Wang et al. found that miR-320 was upregulated in MMVECs in type 2 diabetic Goto-Kakizaki (GK) rats by using miRNA microarray 104. The upregulation of miR-320 decreased the expression of Insulin-like Growth Factor 1 (IGF-1), which could promote angiogenesis, and finally decreased the angiogenic response to diabetic microvascular injury 105.

Interestingly, it was reported that the anti-angiogenic effect of miR-320 on endothelial cells could also be mediated by exosomes from cardiomyocytes 106. Wang et al. observed that cardiomyocytes isolated from type 2 diabetic rats elicited inhibitory effects on angiogenesis through the exosomal transfer of miR-320 into endothelial cells.

In addition to MMVECs, Feng et al. showed that miR-320 could regulate glucose-induced gene expression in human umbilical vein endothelial cells (HUVECs), including Endothelin-1 (ET-1), Vascular Endothelial Growth Factor (VEGF) and Fibronectin (FN), which also participate in angiogenesis 107. Furthermore, Gao et al. found that the miR-320/VEGFA axis was crucial to high-glucose-induced metabolic memory during HUVEC dysfunction and might be involved in the pathology of diabetes 108. In the hyperlipidemic state, oxidative low-density lipoprotein (ox-LDL) induces endothelial dysfunction and apoptosis by increasing oxidative stress in endothelial cells 109. Recently, Xu et al. suggested the lncRNA XIST silencing alleviated EC injury via regulation of the miR-320/NOD2 axis 110.

**Figure 4:**
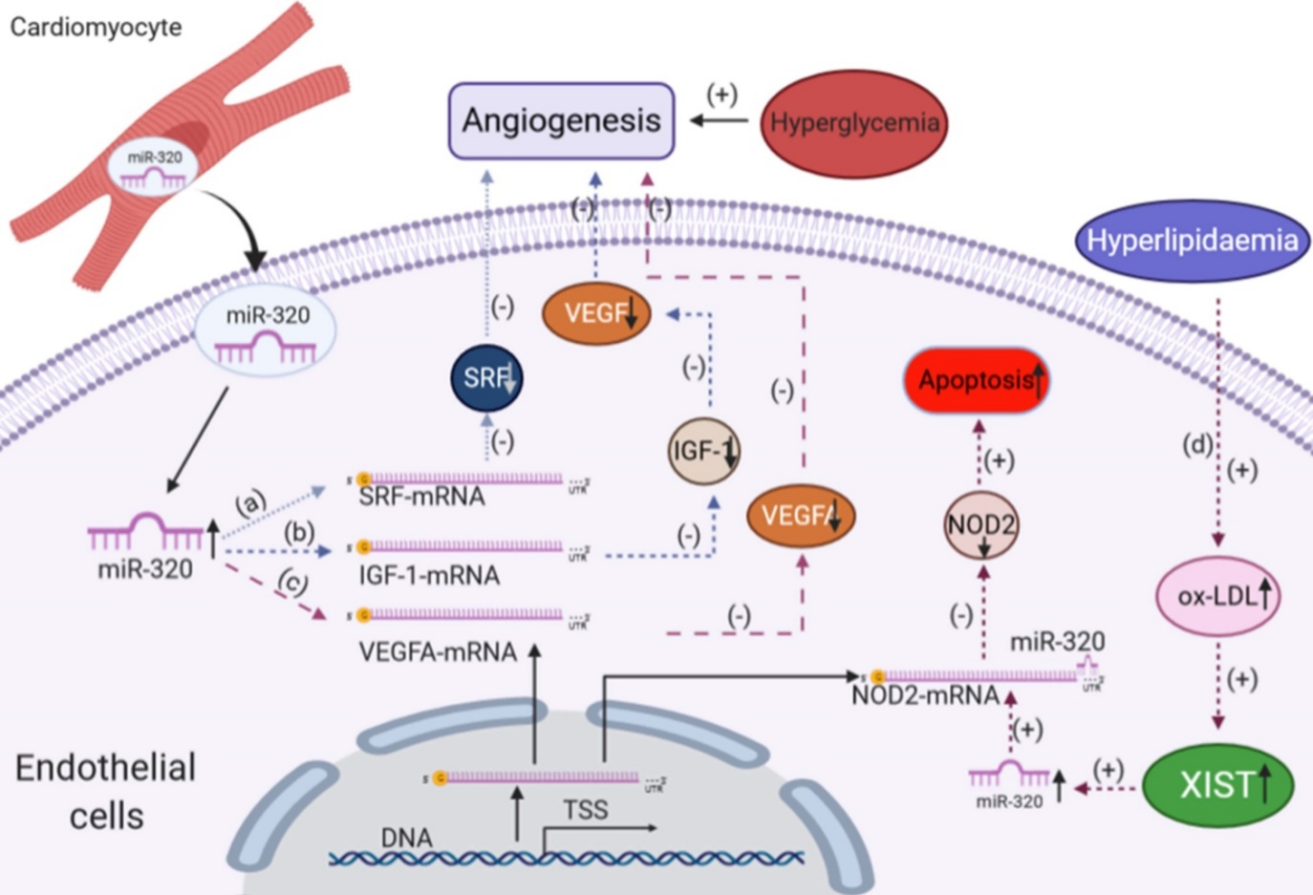
The effects of miR-320 on glucose and lipid metabolism in endothelial cells. (a) In endothelial cells, miR-320 decreased the expression of the SRF gene, leading to the inhibition of angiogenesis. (b) miR-320, derived from cardiomyocytes via exosomes, decreased the expression of the IGF-1 gene, leading to the downregulation of VEGF expression to inhibit angiogenesis. (c) miR-320 decreased the expression of VEGFA to inhibit angiogenesis in the case of hyperglycemia. (d) In the hyperlipidemic state, lncRNA XIST and miR-320 inhibit the protein level of NOD2 leading to apoptosis of endothelial cells. SRF, serum response factor; VEGF, vascular endothelial growth factor; VEGFA, vascular endothelial growth factor A; NOD2, nucleotide binding oligomerization domains; MMVECs, myocardial microvascular endothelial cells; HUVECs, human umbilical vein endothelial cells.

In conclusion, miR-320 also impairs endothelial cell function in diabetic cardiomyopathy, which might provide a new therapeutic strategy for the treatment (Figure 4).

#### The effects of miR-320 on glucose and lipid metabolism in cancer cells

Either under normal physiological or pathological conditions, glucose can be converted into pyruvate by the glycolytic pathway to maintain the normal physiological metabolism of cells 111. Metabolic abnormalities exist in a lot of different cancers including solid tumors of the liver, lung, pancreas, skin, stomach, and uterus as well as acute lymphocytic and myeloid leukemias 112. Abnormal tumor metabolism may be caused by the genetic alterations acquired by tumors modifying their biochemical pathways and tumor dynamic microenvironment 113. It has been reported that tumor cells showed higher rates of glycolysis known as the "Warburg effect", lactate production, changes in lipid synthesis and oxidation occur due to mitochondrial respiration injury and hypoxia, which were frequently associated with the resistance to therapeutic agents 114, compared with corresponding normal tissues 115. For example, Nieman et al. showed that ovarian cancer cells induced fat cell-driven lipolysis and increased the availability of lipids for uptake to rapid division 116. Gastric cancer exhibited a Warburg effect which involving an enhanced uptake of glucose and glycolysis to provide energy under aerobic conditions 117. It is worth mentioning that increased risk of metabolic syndrome was observed in acute lymphoblastic leukemia (ALL) survivors compared with general population 118, 119. Moreover, Poulain et al. showed that AML cells promoted glycolysis and led to glucose addiction to rapid division by constantly over activating mTORC1 signaling 120.

Studies have reported that miR-320 was downregulated in various types of cancers 121-123. As a negative regulator, the overexpression of miR-320 inhibited the proliferation and differentiation of a variety of cancer cells, which in turn suppressed the development and progression of cancer 124, 125. Importantly, miR-320 might also influence the development of cancer by regulating glucose and lipid metabolism. Tang et al. showed that miR-320 played an essential role in the glycolytic pathway during mitochondrial oxidative stress in lung cancer 126. They found that Ets1 (ETS proto-oncogene 1), a transcription factor, could bind to the promoter of hsa-miR-320a in response to H_2_O_2_ treatment, thereby negatively regulate the transcription of miR-320 in several cell types 126. As the expression of miR-320 decreased, the level of glycolytic enzyme muscle-type phosphofructokinase (PFKm) protein, a crucial rate-limiting enzyme of glycolysis, was elevated, thus enhancing the glycolysis process in lung cancer 127.

In addition to glucose metabolism, some studies showed that miR-320 could inhibit the proliferation and differentiation of cancer cells by affecting lipid metabolism. For example, Lei et al. found that miRNA-320 inhibited non-small cell lung cancer cell proliferation, migration, and invasion by targeting fatty acid synthase 128. Tang et al. suggested that miRNA-320 also inhibited osteosarcoma cell proliferation by directly targeting fatty acid synthase 129. Fatty acid synthase, which is highly expressed in most human carcinomas, is a key lipogenic enzyme that catalyzes the terminal steps in the *de novo* biogenesis of fatty acids in cancer pathogenesis 130 and plays critical roles in cancer proliferation and metastasis 131. miR-320 inhibited the fatty acid synthase directly, which in turn reduced the *de novo* biogenesis of fatty acids in cancer cells and finally suppressed cancer cell proliferation, migration, and invasion.

Exosomes are naturally released from living cells and have been well recognized to play critical roles in mediating cell-to-cell communication 132. It is worth mentioning that the current studies about the exosomal transfer of miR-320 mainly focus on cancers 133-135. For example, Stevic et al. determined the miRNA profiles in exosomes from plasma of 106 epithelial ovarian cancer patients, 8 ovarian cystadenoma patients, and 29 healthy women, and they found that miR-320 was significantly enriched in the exosomes from plasma of epithelial ovarian cancer patients as compared to those of healthy women 133. Wan et al. detected that exosomal transfer of miR-320 from leukemia cells to bone marrow mesenchymal stromal cells was an important mediator of leukemia progression and might be a potential therapeutic target for CML 135. Moreover, Manterola et al. demonstrated that miR-320 in the exosomes isolated from the serum of glioblastoma multiforme patients could serve as a potential diagnostic biomarker for glioblastoma multiforme 136. However, the clinical and laboratory data about the exosomal transfer of miR-320 in glucose and lipid metabolism-associated diseases are still limited.

**Figure 5:**
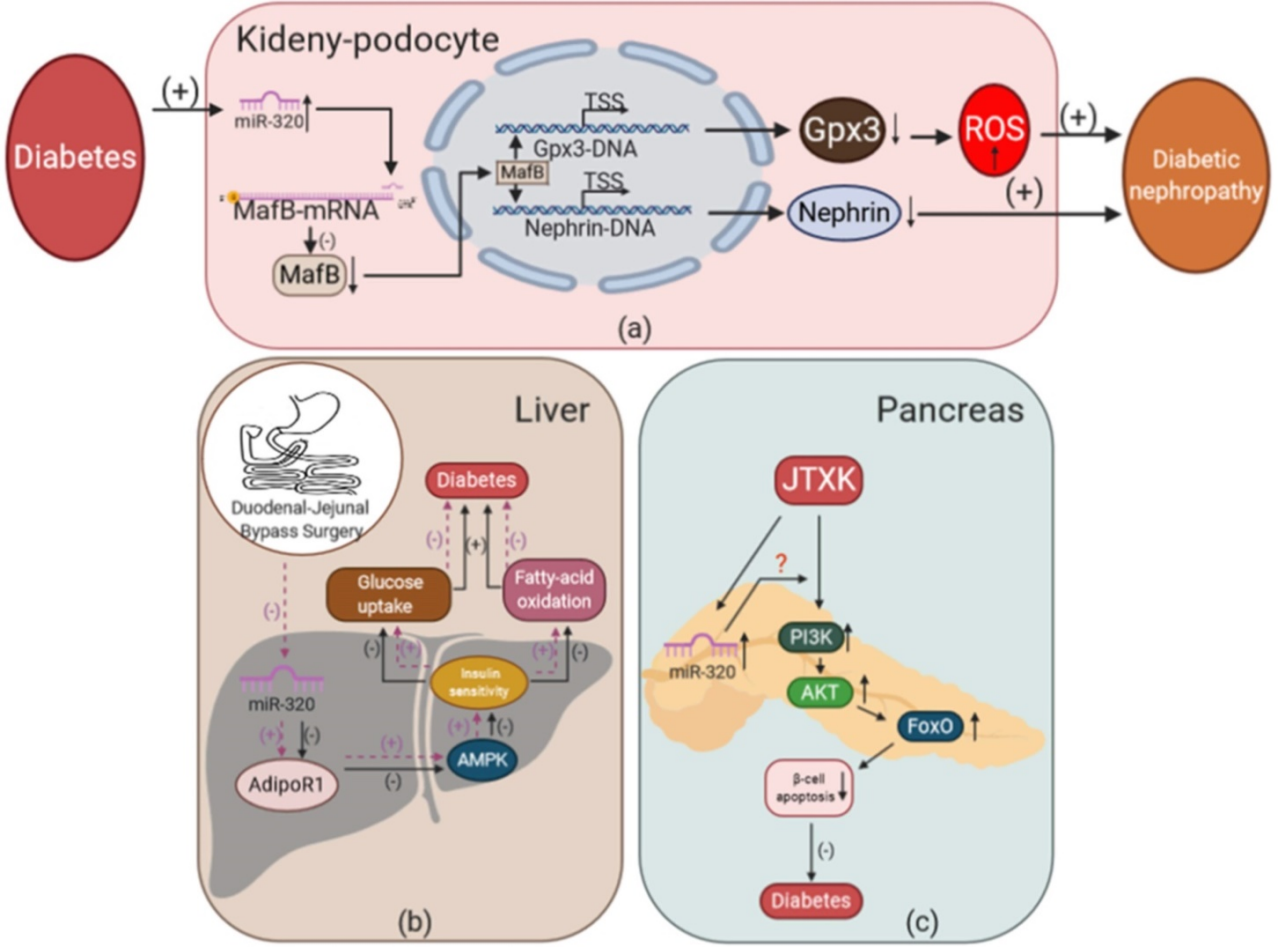
The effects of miR-320 on glucose and lipid metabolism in kidney, liver, and pancreas during diabetes. (a) Overexpression of miR-320 mediated by diabetes could inhibit the expression of MafB to reduce the level of Nephrin and Gpx3 and promote the development of DN. (b) Duodenal-jejunal bypass surgery inhibits the expression of miR-320 in the liver and increases AdipoR1 levels to promote insulin sensitivity via activation of AMPK, leading to increased glucose uptake and fatty acid oxidation, which alleviates the development of diabetes. (c) JTXK could increase the level of miR-320, thereby mediating the effect of PI3K-Akt and FoxO signaling to inhibit the apoptosis of β-cells and exert anti-diabetic effects in the pancreas. Gpx3, glutathione peroxidase 3; AMPK, adenosine monophosphate-activated protein kinase; AdipoR1, adiponectin receptor 1; FoxO, forkhead box class O; JTXK granule, Jiang Tang Xiao Ke granule.

In summary, miR-320 played important roles in regulating glucose and lipid metabolism in various cancer cells, specifically by depriving the energy supply (Figure 5).

### The effects of miR-320 on glucose and lipid metabolism in different target organs

Diabetes is associated with microvascular and macrovascular complications as a result of disordered glucose and lipid metabolism 29. These complications can lead to impaired function of other organs including the heart, liver and kidney through systemic metabolic disorders as described above 29. We are reviewing the effects of miR-320 on glucose and lipid metabolism in the kidney, liver, and pancreas during diabetes.

#### The effects of miR-320 on glucose and lipid metabolism in the kidney during diabetes

To study the effects of miR-320 on glucose and lipid metabolism in the kidney, Chen et al. used a miRNA microarray assay to measure the expression profile of miRNAs in a mouse model of early diabetic nephropathy (DN) 137. They found that miR-320 was increased significantly in the DN mice. Moreover, our lab also showed that miR-320 increased ROS production and induced podocyte apoptosis to promote the development of DN 21. In the kidneys of diabetic mice, we detected that the level of miR-320 was increased. Overexpression of miR-320a could aggravate renal disfunction in DN by targeting MafB and downregulating Nephrin, a key component of the glomerular slit diaphragm that is critical in preventing proteinuria, and Gpx3, an antioxidative stress enzyme in podocytes that increases ROS production 138-140.

#### The effects of miR-320 on glucose and lipid metabolism in the liver during diabetes

In addition to medication such as insulin and antidiabetic drugs, surgery is an important treatment strategy for diabetes. For example, duodenal-jejunal bypass (DJB) has been shown to be an effective surgical treatment for T2DM 141. Meanwhile, miR-320 might mediate the therapeutic effects of DJB in T2DM. Wei et al. found that 5 miRNAs including miR-320 were downregulated significantly in the liver after DJB 142. Further, they found that miR-320 could target AdipoR1, which regulated glucose and fatty acid metabolism partly via the activation of adenosine monophosphate-activated protein kinase (AMPK) 143-145. This suggested that DJB could reduce the expression of miR-320 and increase AdipoR1, which regulated hepatic insulin sensitivity to improve T2DM. Significantly, their study directly showed that the abnormal expression of miR-320 could lead to the disorder of glucose and lipid metabolism in the liver, thus promoting the development of diabetes.

#### The effects of miR-320 on glucose and lipid metabolism in the pancreas during diabetes

A decrease in the number of functional insulin-producing beta cells is the key factor in the development of T2DM and hyperglycemia, while dyslipidemia, cytokines, leptin, autoimmunity, and some sulfonylureas may contribute to the damage of beta cells 146. Interestingly, Mo et al. found that miR-320 was one of the targets of Jiang Tang Xiao Ke (JTXK) granules in mouse pancreatic tissue 147. JTXK is a Chinese herbal formula that has been used clinically to treat T2DM for decades 148. Using miRNA microarray assays, the expression of miR-320 was found to be changed in pancreatic tissue, and miR-320 was predicted to mediate the anti-diabetic effect of JTXK via PI3K-Akt and the FoxO signaling pathway. Though this provided a preliminary strategy to improve the function of beta cells using Chinese herbal medicine, the detailed mechanism remains to be further studied.

**Figure 6:**
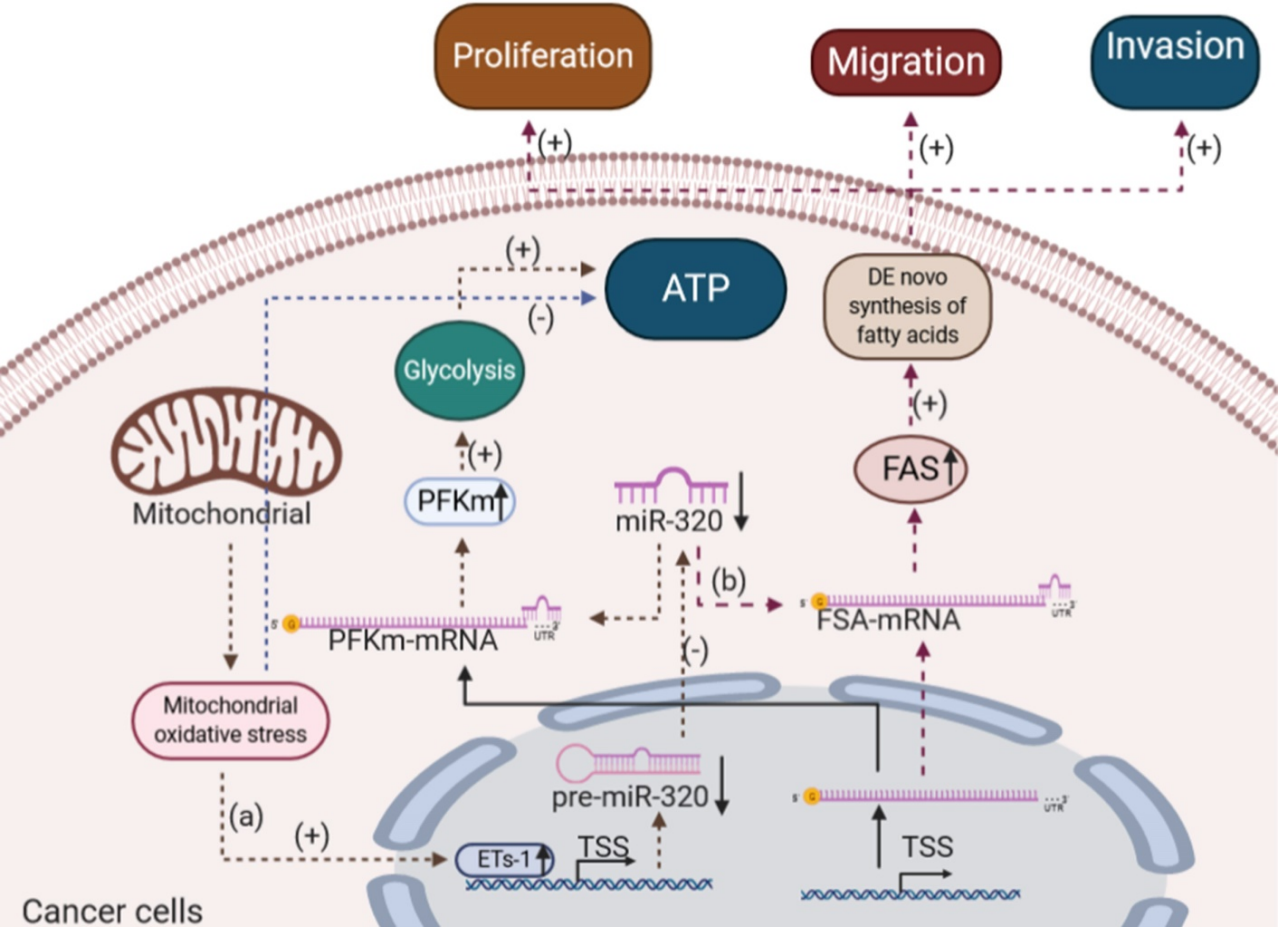
The effects of miR-320 on glucose and lipid metabolism in cancer cells. (a) In lung adenocarcinoma, mitochondrial oxidative stress induces ETs-1, which inhibits the expression of miR-320 and increases the level of PFKm. Thus, the process of glycolysis was strengthened in lung cancer. (b) miR-320 inhibits the FAS level directly to reduce the *de novo* biogenesis of fatty acids in osteosarcoma and non-small cell lung cancer, thus suppressing cancer cell proliferation, migration and invasion. ETs-1, ETS proto-oncogene 1; PFKm, muscle-type phosphofructokinase; FAS, fatty acid synthase.

In conclusion, miR-320 could participate in multiple organ damage in glucose and lipid metabolism disorder-associated diseases, which provides a potential “multiple-effects” therapeutic target of these diseases (Figure 6).

## Conclusion

Although glucose and lipid metabolism disorder-associated diseases have been recognized for many years, the effective prevention and treatment strategies remain elusive. In this article, we first reviewed that miR-320 might be a potential biomarker for metabolic diseases, such as obesity, diabetes, and its complications, which provided a new diagnostic strategy for these diseases. Then, we systematically described the effects of miR-320 on glucose and lipid metabolism disorder-associated diseases. For clinical application, multiple studies suggested that the aberrant expression of miRNAs played essential roles in various diseases, which provided a new therapeutic strategy 149. Here, we reviewed the diagnostic and therapeutic strategies for glucose and lipid metabolism disorder-associated diseases by targeting miR-320. Moreover, we provided a summary table to better illustrate the various miRNAs indicated in the text (Table 1).

However, there are some limitations in the current studies. First, the physiological roles of miR-320 under normal conditions in various organs are missing. Second, the effect of miR-320 on glucose and lipid metabolism is not investigated by the mutual regulation of multiple organs. Furthermore, certain organs are composed of a variety of cell types, and different cell types may interact with each other by exosomes and other vesicles. Considering that the glucose and lipid metabolism disorder-associated diseases are systemic diseases, we should also pay attention to the interaction of miR-320 among certain organs and cell types. Third, studies have reported the roles of miR-320 in various disease conditions using animal models, for example, I/R injury, but the expression patterns of miR-320 in related patients were less investigated, which limited the clinical prospect. Finally, the mechanisms and functions of miR-320 were mainly investigated using expression vectors rather than genetically modified animal models. Although studies using miR-320 transgenic mice have been reported 150, up to now, the studies using miR-320 genetic knockout animal models have not been reported, which might be partially due to side effects on POLR3D. As reported previously, miR-320 is encoded directly upstream of the cell cycle gene POLR3D, which is a conserved subunit specific to RNA polymerase III and affects the overall function of the cell 19, 20. It is worth noting that these models may be achieved by CRISPR-Cas9 or single base site-directed mutations, without affecting the function of POLR3D.

## Figures and Tables

**Table 1:** The expression changes of miRNAs in various metabolic associated-diseases.

miRNAs	Diseases	Objectives	Changes	References
miR-320	CHD	CHD patients	Upregulated	J Cell Mol Med. 2015;19:970
	Atherosclerosis	OPG^-/-^ mouse	Downregulated	PLoS One. 2017;12(3):e0174138
	Diabetic cardiomyopathy	db/db mice	Upregulated	Circ Res. 2019;125:1106
	Diabetic nephropathy	db/db mice	Upregulated	Aging. 2019;11:3055
	Diabetes	T2DM patients	Downregulated	Circ Res. 2010;107(6):810
	Diabetes	T2DM patients	Upregulated	J Clin Endocrinol Metab. 2012;97:E2271
	Diabetes	insulin-resistant 3T3-L1 adipocytes	Upregulated	Clin Exp Pharmacol Physiol. 2009;36:e32
	Obesity	T2DM patients	Upregulated	PLoS One. 2013;8:e77251
	Non-alcoholic fatty liver disease	CCl_4_ treated rats	Upregulated	Toxicol Sci. 2018;166:228
	I/R injury	I/R mice	Upregulated	Cell Mol Biol Lett. 2018;23:41
	I/R injury	myocardial I/R injury rats	Upregulated	Int J Mol Sci. 2014;15:17442
	I/R injury	ischemic H9c2 cardiomyocytes	Upregulated	Cell Biochem Funct. 2018;36:166
	Diabetes	type 2 diabetic GK rats	Upregulated	Arterioscler Thromb Vasc Biol. 2004;24:435
	Diabetic cardiomyopathy	type 2 diabetic GK rats	Upregulated	Clin Exp Pharmacol Physiol. 2009;36:181
	Diabetic cardiomyopathy	type 2 diabetic GK rats	Upregulated	J Mol Cell Cardiol. 2014;74:139
	Diabetes	high glucose treated with HUVECs	Upregulated	ISRN Endocrinol. 2012;2012:549875, Microvasc Res. 2020;127:103913
	Hyperlipidemic	ox-LDL treated with HUVECs	Upregulated	Biochem Biophys Res Commun. 2018;503:586
	Diabetic nephropathy	STZ treated mice	Upregulated	J Nephrol. 2012;25:566
	Diabetes	DJB rats	Downregulated	Surg Obes Relat Dis. 2018;14:960
	T2DM and hyperglycemia	KKAy diabetic mice	Upregulated	Front Pharmacol. 2017;8:795
	Lung cancer	human lung adenocarcinoma tissues	Downregulated	FASEB J. 2012;26:4710
	Non-small cell lung cancer	human NSCLC cell lines	Downregulated	Mol Med Rep. 2016;14:1255
	Osteosarcoma	human osteosarcoma tissues	Downregulated	Tumour Biol. 2014;35:4177
miR-320e	CHD	CHD patients	Upregulated	J Cell Mol Med. 2020;24(11):5984-5997.
miRNA-425-3p, miRNA-933, miRNA-191, miR-320e, miR-320b	Hyperlipidemia	Hyperlipidemia patients	Upregulated	Lipids Health Dis. 2019;18:104
miR-206	Hyperlipidemia	High-fat diet treated C57Bl/6J mice	Downregulated	J Hepatol. 2017;66:816
	CHD	CHD patients	Upregulated	Biosci Rep. 2015;36:e00295
miR-574-5p	CHD	CHD patients	Upregulated	Biosci Rep. 2015;36:e00295
MiR-92a	Diabetes	Min-6 mouse pancreatic b-cell	Downregulated	Biochem Biophys Res Commun. 2018;500:577
miR-142-3p	CHD	CHD patients	Upregulated	Medicine (Baltimore). 2018;97:e11428
miR-17-5p	CHD	CHD patients	Upregulated	Medicine. 2018;97:e11428
miR-30e	Atherosclerosis	coronary atherosclerosis patients	Upregulated	Mol Med Rep. 2019;19:3298
miR-138, miR-503, miR-376a	Obesity	T2DM patients	Upregulated	PLoS One. 2013;8:e77251

CHD, Coronary heart disease; T2DM, type 2 diabetes; I/R, ischemia-reperfusion injury; HUVECs, Human Umbilical Vein Endothelial Cells; ox-LDL, oxidative low-density lipoprotein; STZ, Streptozocin; DJB, Duodenal-jejunal bypass; NSCLC, non‑small cell lung cancer.

## References

[1] Aguilar M, Bhuket T, Torres S, Liu B, Wong RJ (2015). Prevalence of the metabolic syndrome in the United States, 2003-2012. JAMA.

[2] Kurylowicz A, Cakala-Jakimowicz M, Puzianowska-Kuznicka M (2020). Targeting Abdominal Obesity and Its Complications with Dietary Phytoestrogens. Nutrients.

[3] Ya L, Jie Y, Mengxue Y, Pan W, Changwei Y, Jie X (2019). Identification of Nonylphenol and Glucolipid Metabolism-Related Proteins in the Serum of Type 2 Diabetes Patients. Iran J Public Health.

[4] Zhu ZY, Wang F, Jia CH, Xie ML (2019). Apigenin-induced HIF-1alpha inhibitory effect improves abnormal glucolipid metabolism in Ang/hypoxia-stimulated or HIF-1alpha-overexpressed H9c2 cells. Phytomedicine.

[5] Wang F, Liu HC, Liu XS, Dong SN, Pan D, Yang LG (2019). [Effects of omega-3 polyunsaturated fatty acids from different sources on glucolipid metabolism in type 2 diabetic patients with dyslipidemia]. Zhonghua Yu Fang Yi Xue Za Zhi.

[6] Zhao L, Cang Z, Sun H, Nie X, Wang N, Lu Y (2017). Berberine improves glucogenesis and lipid metabolism in nonalcoholic fatty liver disease. BMC Endocr Disord.

[7] Gao X, Mi S, Zhang F, Gong F, Lai Y, Gao F (2011). Association of chemerin mRNA expression in human epicardial adipose tissue with coronary atherosclerosis. Cardiovasc Diabetol.

[8] Filla LA, Edwards JL (2016). Metabolomics in diabetic complications. Mol Biosyst.

[9] American Diabetes A (2011). Diagnosis and classification of diabetes mellitus. Diabetes Care.

[10] Bartel DP (2004). MicroRNAs: genomics, biogenesis, mechanism, and function. Cell.

[11] Qadir MI, Faheem A (2017). miRNA: A Diagnostic and Therapeutic Tool for Pancreatic Cancer. Crit Rev Eukaryot Gene Expr.

[12] Xu J, Chen Z, Wang Y, Wang X, Chen L, Yuan T (2019). Several circulating miRNAs related to hyperlipidemia and atherosclerotic cardiovascular diseases. Lipids Health Dis.

[13] Satake E, Pezzolesi MG, Md Dom ZI, Smiles AM, Niewczas MA, Krolewski AS (2018). Circulating miRNA Profiles Associated With Hyperglycemia in Patients With Type 1 Diabetes. Diabetes.

[14] Silambarasan M, Tan JR, Karolina DS, Armugam A, Kaur C, Jeyaseelan K (2016). MicroRNAs in Hyperglycemia Induced Endothelial Cell Dysfunction. Int J Mol Sci.

[15] Karolina DS, Armugam A, Tavintharan S, Wong MT, Lim SC, Sum CF (2011). MicroRNA 144 impairs insulin signaling by inhibiting the expression of insulin receptor substrate 1 in type 2 diabetes mellitus. PLoS One.

[16] Chen C, Wang Y, Yang S, Li H, Zhao G, Wang F (2015). MiR-320a contributes to atherogenesis by augmenting multiple risk factors and down-regulating SRF. J Cell Mol Med.

[17] Li H, Fan J, Zhao Y, Zhang X, Dai B, Zhan J (2019). Nuclear miR-320 Mediates Diabetes-Induced Cardiac Dysfunction by Activating Transcription of Fatty Acid Metabolic Genes to Cause Lipotoxicity in the Heart. Circ Res.

[18] McCreight JC, Schneider SE, Wilburn DB, Swanson WJ (2017). Evolution of microRNA in primates. PLoS One.

[19] Kim DH, Saetrom P, Snove O Jr, Rossi JJ (2008). MicroRNA-directed transcriptional gene silencing in mammalian cells. Proc Natl Acad Sci U S A.

[20] Kato M, Castro NE, Natarajan R (2013). MicroRNAs: potential mediators and biomarkers of diabetic complications. Free Radic Biol Med.

[21] He M, Wang J, Yin Z, Zhao Y, Hou H, Fan J (2019). MiR-320a induces diabetic nephropathy via inhibiting MafB. Aging (Albany NY).

[22] Aparicio-Puerta E, Jaspez D, Lebron R, Koppers-Lalic D, Marchal JA, Hackenberg M (2019). liqDB: a small-RNAseq knowledge discovery database for liquid biopsy studies. Nucleic Acids Res.

[23] Cho WC (2010). MicroRNAs: potential biomarkers for cancer diagnosis, prognosis and targets for therapy. Int J Biochem Cell Biol.

[24] Liu N, Chen NY, Cui RX, Li WF, Li Y, Wei RR (2012). Prognostic value of a microRNA signature in nasopharyngeal carcinoma: a microRNA expression analysis. Lancet Oncol.

[25] Shin VY, Chu KM (2014). MiRNA as potential biomarkers and therapeutic targets for gastric cancer. World J Gastroenterol.

[26] Karolina DS, Tavintharan S, Armugam A, Sepramaniam S, Pek SL, Wong MT (2012). Circulating miRNA profiles in patients with metabolic syndrome. J Clin Endocrinol Metab.

[27] Jimenez-Lucena R, Rangel-Zuniga OA, Alcala-Diaz JF, Lopez-Moreno J, Roncero-Ramos I, Molina-Abril H (2018). Circulating miRNAs as Predictive Biomarkers of Type 2 Diabetes Mellitus Development in Coronary Heart Disease Patients from the CORDIOPREV Study. Mol Ther Nucleic Acids.

[28] Forbes JM, Fotheringham AK (2017). Vascular complications in diabetes: old messages, new thoughts. Diabetologia.

[29] Boudina S, Abel ED (2007). Diabetic cardiomyopathy revisited. Circulation.

[30] Saltiel AR, Kahn CR (2001). Insulin signalling and the regulation of glucose and lipid metabolism. Nature.

[31] Holscher ME, Bode C, Bugger H (2016). Diabetic Cardiomyopathy: Does the Type of Diabetes Matter?. Int J Mol Sci.

[32] Stumvoll M, Goldstein BJ, van Haeften TW (2005). Type 2 diabetes: principles of pathogenesis and therapy. Lancet.

[33] Gillett MJ (2009). International Expert Committee report on the role of the A1c assay in the diagnosis of diabetes: Diabetes Care 2009; 32(7): 1327-1334. Clin Biochem Rev.

[34] Othman A, Saely CH, Muendlein A, Vonbank A, Drexel H, von Eckardstein A (2015). Plasma 1-deoxysphingolipids are predictive biomarkers for type 2 diabetes mellitus. BMJ Open Diabetes Res Care.

[35] Kolberg JA, Jorgensen T, Gerwien RW, Hamren S, McKenna MP, Moler E (2009). Development of a type 2 diabetes risk model from a panel of serum biomarkers from the Inter99 cohort. Diabetes Care.

[36] Guay C, Regazzi R (2013). Circulating microRNAs as novel biomarkers for diabetes mellitus. Nat Rev Endocrinol.

[37] Zampetaki A, Kiechl S, Drozdov I, Willeit P, Mayr U, Prokopi M (2010). Plasma microRNA profiling reveals loss of endothelial miR-126 and other microRNAs in type 2 diabetes. Circ Res.

[38] Kong L, Zhu J, Han W, Jiang X, Xu M, Zhao Y (2011). Significance of serum microRNAs in pre-diabetes and newly diagnosed type 2 diabetes: a clinical study. Acta Diabetol.

[39] Ling HY, Ou HS, Feng SD, Zhang XY, Tuo QH, Chen LX (2009). CHANGES IN microRNA (miR) profile and effects of miR-320 in insulin-resistant 3T3-L1 adipocytes. Clin Exp Pharmacol Physiol.

[40] Katayama M, Wiklander OPB, Fritz T, Caidahl K, El-Andaloussi S, Zierath JR (2019). Circulating Exosomal miR-20b-5p Is Elevated in Type 2 Diabetes and Could Impair Insulin Action in Human Skeletal Muscle. Diabetes.

[41] Yahagi K, Kolodgie FD, Otsuka F, Finn AV, Davis HR, Joner M (2016). Pathophysiology of native coronary, vein graft, and in-stent atherosclerosis. Nat Rev Cardiol.

[42] Libby P, Ridker PM, Hansson GK (2011). Progress and challenges in translating the biology of atherosclerosis. Nature.

[43] Tibaut M, Caprnda M, Kubatka P, Sinkovic A, Valentova V, Filipova S (2019). Markers of Atherosclerosis: Part 2 - Genetic and Imaging Markers. Heart Lung Circ.

[44] Zhang Y, Liu D, Chen X, Li J, Li L, Bian Z (2010). Secreted monocytic miR-150 enhances targeted endothelial cell migration. Mol Cell.

[45] Lu M, Yuan S, Li S, Li L, Liu M, Wan S (2019). The Exosome-Derived Biomarker in Atherosclerosis and Its Clinical Application. J Cardiovasc Transl Res.

[46] Wang Z, Zhang J, Zhang S, Yan S, Wang Z, Wang C (2019). MiR30e and miR92a are related to atherosclerosis by targeting ABCA1. Mol Med Rep.

[47] Wong ND (2014). Epidemiological studies of CHD and the evolution of preventive cardiology. Nat Rev Cardiol.

[48] Mortality GBD, Causes of Death C (2015). Global, regional, and national age-sex specific all-cause and cause-specific mortality for 240 causes of death, 1990-2013: a systematic analysis for the Global Burden of Disease Study 2013. Lancet.

[49] Wang Y, Yu Q, Fan D, Cao F (2012). Coronary heart disease in type 2 diabetes: mechanisms and comprehensive prevention strategies. Expert Rev Cardiovasc Ther.

[50] Sharma A, Arbab-Zadeh A (2012). Assessment of coronary heart disease by CT angiography: current and evolving applications. J Nucl Cardiol.

[51] Genereux P, Mehran R, Leon MB, Bettinger N, Stone GW (2017). Classification for Assessing the Quality of Diagnostic Coronary Angiography. J Invasive Cardiol.

[52] Zhou J, Shao G, Chen X, Yang X, Huang X, Peng P (2015). miRNA 206 and miRNA 574-5p are highly expression in coronary artery disease. Biosci Rep.

[53] Zhong Z, Hou J, Zhang Q, Zhong W, Li B, Li C (2018). Circulating microRNA expression profiling and bioinformatics analysis of dysregulated microRNAs of patients with coronary artery disease. Medicine (Baltimore).

[54] Su M, Niu Y, Dang Q, Qu J, Zhu D, Tang Z (2020). Circulating microRNA profiles based on direct S-Poly(T)Plus assay for detection of coronary heart disease. J Cell Mol Med.

[55] Liu J, Xiao X, Shen Y, Chen L, Xu C, Zhao H (2017). MicroRNA-32 promotes calcification in vascular smooth muscle cells: Implications as a novel marker for coronary artery calcification. PLoS One.

[56] Horrobin DF (1988). The roles of essential fatty acids in the development of diabetic neuropathy and other complications of diabetes mellitus. Prostaglandins Leukot Essent Fatty Acids.

[57] Newfield RS, Dewan AK, Jain S (2008). Dyslipidemia in children with type 2 diabetes vs. obesity. Pediatr Diabetes.

[58] Sellers EA, Yung G, Dean HJ (2007). Dyslipidemia and other cardiovascular risk factors in a Canadian First Nation pediatric population with type 2 diabetes mellitus. Pediatr Diabetes.

[59] Bastien M, Poirier P, Lemieux I, Despres JP (2014). Overview of epidemiology and contribution of obesity to cardiovascular disease. Prog Cardiovasc Dis.

[60] Viesti ACR, Salgado W Jr, Pretti da Cunha Tirapelli D, dos Santos JS (2014). The expression of LEP, LEPR, IGF1 and IL10 in obesity and the relationship with microRNAs. PLoS One.

[61] Heneghan HM, Miller N, McAnena OJ, O'Brien T, Kerin MJ (2011). Differential miRNA expression in omental adipose tissue and in the circulation of obese patients identifies novel metabolic biomarkers. J Clin Endocrinol Metab.

[62] Olumi AF (2014). Commentary on "randomized clinical trial of vitamin D3 doses on prostatic vitamin D metabolite levels and Ki67 labeling in prostate cancer patients." Wagner D, Trudel D, Van der Kwast T, Nonn L, Giangreco AA, Li D, Dias A, Cardoza M, Laszlo S, Hersey K, Klotz L, Finelli A, Fleshner N, Vieth R, Department of Nutritional Sciences, University of Toronto, Ontario, Canada.: J Clin Endocrinol Metab 2013;98(4):1498-507 [Epub 2013 Mar 5]. Urol Oncol.

[63] Pescador N, Perez-Barba M, Ibarra JM, Corbaton A, Martinez-Larrad MT, Serrano-Rios M (2013). Serum circulating microRNA profiling for identification of potential type 2 diabetes and obesity biomarkers. PLoS One.

[64] Munetsuna E, Yamada H, Ando Y, Yamazaki M, Tsuboi Y, Kondo M (2018). Association of subcutaneous and visceral fat with circulating microRNAs in a middle-aged Japanese population. Ann Clin Biochem.

[65] Berlanga A, Guiu-Jurado E, Porras JA, Auguet T (2014). Molecular pathways in non-alcoholic fatty liver disease. Clin Exp Gastroenterol.

[66] Kagawa T, Shirai Y, Oda S, Yokoi T (2018). Identification of Specific MicroRNA Biomarkers in Early Stages of Hepatocellular Injury, Cholestasis, and Steatosis in Rats. Toxicol Sci.

[67] Petersen MC, Shulman GI (2018). Mechanisms of Insulin Action and Insulin Resistance. Physiol Rev.

[68] Leal-Esteban LC, Fajas L (2020). Cell cycle regulators in cancer cell metabolism. Biochim Biophys Acta Mol Basis Dis.

[69] Taniguchi CM, Emanuelli B, Kahn CR (2006). Critical nodes in signalling pathways: insights into insulin action. Nat Rev Mol Cell Biol.

[70] van Dam EM, Govers R, James DE (2005). Akt activation is required at a late stage of insulin-induced GLUT4 translocation to the plasma membrane. Mol Endocrinol.

[71] Salarinasab S, Salimi L, Alidadiani N, Shokrollahi E, Arzhanga P, Karbasforush S (2020). Interaction of opioid with insulin/IGFs signaling in Alzheimer's disease. J Mol Neurosci.

[72] Qiao L, Li Y, Sun S (2020). Insulin Exacerbates Inflammation in Fibroblast-Like Synoviocytes. Inflammation.

[73] Liu L, Li X (2019). Downregulation of miR-320 Alleviates Endoplasmic Reticulum Stress and Inflammatory Response in 3T3-L1 Adipocytes. Exp Clin Endocrinol Diabetes.

[74] So JS (2018). Roles of Endoplasmic Reticulum Stress in Immune Responses. Mol Cells.

[75] Wakabayashi S, Yoshida H (2013). The essential biology of the endoplasmic reticulum stress response for structural and computational biologists. Comput Struct Biotechnol J.

[76] Shi XY, Li JT (2018). [Endoplasmic reticulum stress in regulation of hepatic fibrosis]. Zhonghua Gan Zang Bing Za Zhi.

[77] Ozcan U, Cao Q, Yilmaz E, Lee AH, Iwakoshi NN, Ozdelen E (2004). Endoplasmic reticulum stress links obesity, insulin action, and type 2 diabetes. Science.

[78] Deng J, Liu S, Zou L, Xu C, Geng B, Xu G (2012). Lipolysis response to endoplasmic reticulum stress in adipose cells. J Biol Chem.

[79] Rosca MG, Vazquez EJ, Kerner J, Parland W, Chandler MP, Stanley W (2008). Cardiac mitochondria in heart failure: decrease in respirasomes and oxidative phosphorylation. Cardiovasc Res.

[80] Gertz EW, Wisneski JA, Stanley WC, Neese RA (1988). Myocardial substrate utilization during exercise in humans. Dual carbon-labeled carbohydrate isotope experiments. J Clin Invest.

[81] Stanley WC, Lopaschuk GD, Hall JL, McCormack JG (1997). Regulation of myocardial carbohydrate metabolism under normal and ischaemic conditions. Potential for pharmacological interventions. Cardiovasc Res.

[82] Tian ZQ, Jiang H, Lu ZB (2018). MiR-320 regulates cardiomyocyte apoptosis induced by ischemia-reperfusion injury by targeting AKIP1. Cell Mol Biol Lett.

[83] Yu H, Tigchelaar W, Koonen DP, Patel HH, de Boer RA, van Gilst WH (2013). AKIP1 expression modulates mitochondrial function in rat neonatal cardiomyocytes. PLoS One.

[84] Sastri M, Haushalter KJ, Panneerselvam M, Chang P, Fridolfsson H, Finley JC (2013). A kinase interacting protein (AKIP1) is a key regulator of cardiac stress. Proc Natl Acad Sci U S A.

[85] Yu L, Fan C, Li Z, Zhang J, Xue X, Xu Y (2017). Melatonin rescues cardiac thioredoxin system during ischemia-reperfusion injury in acute hyperglycemic state by restoring Notch1/Hes1/Akt signaling in a membrane receptor-dependent manner. J Pineal Res.

[86] Nagasaka S, Katoh H, Niu CF, Matsui S, Urushida T, Satoh H (2007). Protein kinase A catalytic subunit alters cardiac mitochondrial redox state and membrane potential via the formation of reactive oxygen species. Circ J.

[87] Song CL, Liu B, Diao HY, Shi YF, Li YX, Zhang JC (2014). The protective effect of microRNA-320 on left ventricular remodeling after myocardial ischemia-reperfusion injury in the rat model. Int J Mol Sci.

[88] Song CL, Liu B, Diao HY, Shi YF, Zhang JC, Li YX (2016). Down-regulation of microRNA-320 suppresses cardiomyocyte apoptosis and protects against myocardial ischemia and reperfusion injury by targeting IGF-1. Oncotarget.

[89] Fath-Ordoubadi F, Beatt KJ (1997). Glucose-insulin-potassium therapy for treatment of acute myocardial infarction: an overview of randomized placebo-controlled trials. Circulation.

[90] Malmberg K (1997). Prospective randomised study of intensive insulin treatment on long term survival after acute myocardial infarction in patients with diabetes mellitus. DIGAMI (Diabetes Mellitus, Insulin Glucose Infusion in Acute Myocardial Infarction) Study Group. BMJ.

[91] Yang N, Wu L, Zhao Y, Zou N, Liu C (2018). MicroRNA-320 involves in the cardioprotective effect of insulin against myocardial ischemia by targeting survivin. Cell Biochem Funct.

[92] Altieri DC (2008). Survivin, cancer networks and pathway-directed drug discovery. Nat Rev Cancer.

[93] Altieri DC (2010). Survivin and IAP proteins in cell-death mechanisms. Biochem J.

[94] Tao N, Wagner SJ, Lublin DM (1996). CD36 is palmitoylated on both N- and C-terminal cytoplasmic tails. J Biol Chem.

[95] Li H, Zhan J, Zhao Y, Fan J, Yuan S, Yin Z (2020). Identification of ncRNA-Mediated Functions of Nucleus-Localized miR-320 in Cardiomyocytes. Mol Ther Nucleic Acids.

[96] Cai L, Wang Y, Zhou G, Chen T, Song Y, Li X (2006). Attenuation by metallothionein of early cardiac cell death via suppression of mitochondrial oxidative stress results in a prevention of diabetic cardiomyopathy. J Am Coll Cardiol.

[97] Cai L (2006). Suppression of nitrative damage by metallothionein in diabetic heart contributes to the prevention of cardiomyopathy. Free Radic Biol Med.

[98] Finck BN, Lehman JJ, Leone TC, Welch MJ, Bennett MJ, Kovacs A (2002). The cardiac phenotype induced by PPARalpha overexpression mimics that caused by diabetes mellitus. J Clin Invest.

[99] Finck BN, Han X, Courtois M, Aimond F, Nerbonne JM, Kovacs A (2003). A critical role for PPARalpha-mediated lipotoxicity in the pathogenesis of diabetic cardiomyopathy: modulation by dietary fat content. Proc Natl Acad Sci U S A.

[100] Okada H, Woodcock-Mitchell J, Mitchell J, Sakamoto T, Marutsuka K, Sobel BE (1998). Induction of plasminogen activator inhibitor type 1 and type 1 collagen expression in rat cardiac microvascular endothelial cells by interleukin-1 and its dependence on oxygen-centered free radicals. Circulation.

[101] Okruhlicova L, Tribulova N, Weismann P, Sotnikova R (2005). Ultrastructure and histochemistry of rat myocardial capillary endothelial cells in response to diabetes and hypertension. Cell Res.

[102] Farhangkhoee H, Khan ZA, Kaur H, Xin X, Chen S, Chakrabarti S (2006). Vascular endothelial dysfunction in diabetic cardiomyopathy: pathogenesis and potential treatment targets. Pharmacol Ther.

[103] Waltenberger J (2001). Impaired collateral vessel development in diabetes: potential cellular mechanisms and therapeutic implications. Cardiovasc Res.

[104] Delafontaine P, Song YH, Li Y (2004). Expression, regulation, and function of IGF-1, IGF-1R, and IGF-1 binding proteins in blood vessels. Arterioscler Thromb Vasc Biol.

[105] Wang XH, Qian RZ, Zhang W, Chen SF, Jin HM, Hu RM (2009). MicroRNA-320 expression in myocardial microvascular endothelial cells and its relationship with insulin-like growth factor-1 in type 2 diabetic rats. Clin Exp Pharmacol Physiol.

[106] Wang X, Huang W, Liu G, Cai W, Millard RW, Wang Y (2014). Cardiomyocytes mediate anti-angiogenesis in type 2 diabetic rats through the exosomal transfer of miR-320 into endothelial cells. J Mol Cell Cardiol.

[107] Feng B, Chakrabarti S (2012). miR-320 Regulates Glucose-Induced Gene Expression in Diabetes. ISRN Endocrinol.

[108] Gao J, Ailifeire M, Wang C, Luo L, Zhang J, Yuan L (2020). miR-320/VEGFA axis affects high glucose-induced metabolic memory during human umbilical vein endothelial cell dysfunction in diabetes pathology. Microvasc Res.

[109] Pandey D, Bhunia A, Oh YJ, Chang F, Bergman Y, Kim JH (2014). OxLDL triggers retrograde translocation of arginase2 in aortic endothelial cells via ROCK and mitochondrial processing peptidase. Circ Res.

[110] Xu X, Ma C, Liu C, Duan Z, Zhang L (2018). Knockdown of long noncoding RNA XIST alleviates oxidative low-density lipoprotein-mediated endothelial cells injury through modulation of miR-320/NOD2 axis. Biochem Biophys Res Commun.

[111] Heim P, Morandi C, Brouwer GR, Xu L, Montessuit C, Brink M (2020). Neuregulin-1 triggers GLUT4 translocation and enhances glucose uptake independently of insulin receptor substrate and ErbB3 in neonatal rat cardiomyocytes. Biochim Biophys Acta Mol Cell Res.

[112] Semenza GL (2010). Defining the role of hypoxia-inducible factor 1 in cancer biology and therapeutics. Oncogene.

[113] He Q, Xu RZ, Shkarin P, Pizzorno G, Lee-French CH, Rothman DL (2003). Magnetic resonance spectroscopic imaging of tumor metabolic markers for cancer diagnosis, metabolic phenotyping, and characterization of tumor microenvironment. Dis Markers.

[114] Xu RH, Pelicano H, Zhou Y, Carew JS, Feng L, Bhalla KN (2005). Inhibition of glycolysis in cancer cells: a novel strategy to overcome drug resistance associated with mitochondrial respiratory defect and hypoxia. Cancer Res.

[115] Jones RG, Thompson CB (2009). Tumor suppressors and cell metabolism: a recipe for cancer growth. Genes Dev.

[116] Nieman KM, Kenny HA, Penicka CV, Ladanyi A, Buell-Gutbrod R, Zillhardt MR (2011). Adipocytes promote ovarian cancer metastasis and provide energy for rapid tumor growth. Nat Med.

[117] Liberti MV, Locasale JW (2016). The Warburg Effect: How Does it Benefit Cancer Cells?. Trends Biochem Sci.

[118] Talvensaari KK, Lanning M, Tapanainen P, Knip M (1996). Long-term survivors of childhood cancer have an increased risk of manifesting the metabolic syndrome. J Clin Endocrinol Metab.

[119] Nuver J, Smit AJ, Postma A, Sleijfer DT, Gietema JA (2002). The metabolic syndrome in long-term cancer survivors, an important target for secondary preventive measures. Cancer Treat Rev.

[120] Poulain L, Sujobert P, Zylbersztejn F, Barreau S, Stuani L, Lambert M (2017). High mTORC1 activity drives glycolysis addiction and sensitivity to G6PD inhibition in acute myeloid leukemia cells. Leukemia.

[121] Schaar DG, Medina DJ, Moore DF, Strair RK, Ting Y (2009). miR-320 targets transferrin receptor 1 (CD71) and inhibits cell proliferation. Exp Hematol.

[122] Schepeler T, Reinert JT, Ostenfeld MS, Christensen LL, Silahtaroglu AN, Dyrskjot L (2008). Diagnostic and prognostic microRNAs in stage II colon cancer. Cancer Res.

[123] Hsieh IS, Chang KC, Tsai YT, Ke JY, Lu PJ, Lee KH (2013). MicroRNA-320 suppresses the stem cell-like characteristics of prostate cancer cells by downregulating the Wnt/beta-catenin signaling pathway. Carcinogenesis.

[124] Luo L, Yang R, Zhao S, Chen Y, Hong S, Wang K (2018). Decreased miR-320 expression is associated with breast cancer progression, cell migration, and invasiveness via targeting Aquaporin 1. Acta Biochim Biophys Sin (Shanghai).

[125] Pan C, Gao H, Zheng N, Gao Q, Si Y, Zhao Y (2017). MiR-320 inhibits the growth of glioma cells through downregulating PBX3. Biol Res.

[126] Tang H, Lee M, Sharpe O, Salamone L, Noonan EJ, Hoang CD (2012). Oxidative stress-responsive microRNA-320 regulates glycolysis in diverse biological systems. FASEB J.

[127] Mulukutla BC, Yongky A, Daoutidis P, Hu WS (2014). Bistability in glycolysis pathway as a physiological switch in energy metabolism. PLoS One.

[128] Lei T, Zhu Y, Jiang C, Wang Y, Fu J, Fan Z (2016). MicroRNA-320 was downregulated in non-small cell lung cancer and inhibited cell proliferation, migration and invasion by targeting fatty acid synthase. Mol Med Rep.

[129] Cheng C, Chen ZQ, Shi XT (2014). MicroRNA-320 inhibits osteosarcoma cells proliferation by directly targeting fatty acid synthase. Tumour Biol.

[130] Menendez JA, Lupu R (2007). Fatty acid synthase and the lipogenic phenotype in cancer pathogenesis. Nat Rev Cancer.

[131] Zaytseva YY, Rychahou PG, Gulhati P, Elliott VA, Mustain WC, O'Connor K (2012). Inhibition of fatty acid synthase attenuates CD44-associated signaling and reduces metastasis in colorectal cancer. Cancer Res.

[132] Koritzinsky EH, Street JM, Star RA, Yuen PS (2017). Quantification of Exosomes. J Cell Physiol.

[133] Pan C, Stevic I, Muller V, Ni Q, Oliveira-Ferrer L, Pantel K (2018). Exosomal microRNAs as tumor markers in epithelial ovarian cancer. Mol Oncol.

[134] Beuzelin D, Pitard B, Kaeffer B (2019). Oral Delivery of miRNA With Lipidic Aminoglycoside Derivatives in the Breastfed Rat. Front Physiol.

[135] Gao X, Wan Z, Wei M, Dong Y, Zhao Y, Chen X (2019). Chronic myelogenous leukemia cells remodel the bone marrow niche via exosome-mediated transfer of miR-320. Theranostics.

[136] Manterola L, Guruceaga E, Gallego Perez-Larraya J, Gonzalez-Huarriz M, Jauregui P, Tejada S (2014). A small noncoding RNA signature found in exosomes of GBM patient serum as a diagnostic tool. Neuro Oncol.

[137] Chen YQ, Wang XX, Yao XM, Zhang DL, Yang XF, Tian SF (2012). Abated microRNA-195 expression protected mesangial cells from apoptosis in early diabetic renal injury in mice. J Nephrol.

[138] Bonnet F, Tikellis C, Kawachi H, Burns WC, Wookey PJ, Cao Z (2002). Nephrin expression in the post-natal developing kidney in normotensive and hypertensive rats. Clin Exp Hypertens.

[139] Shirota S, Yoshida T, Sakai M, Kim JI, Sugiura H, Oishi T (2006). Correlation between the expression level of c-maf and glutathione peroxidase-3 in c-maf -/- mice kidney and c-maf overexpressed renal tubular cells. Biochem Biophys Res Commun.

[140] Jefferson JA, Shankland SJ, Pichler RH (2008). Proteinuria in diabetic kidney disease: a mechanistic viewpoint. Kidney Int.

[141] Li M, Li H, Zhou Z, Zhou Y, Wang Y, Zhang X (2016). Duodenal-Jejunal Bypass Surgery Ameliorates Glucose Homeostasis and Reduces Endoplasmic Reticulum Stress in the Liver Tissue in a Diabetic Rat Model. Obes Surg.

[142] Wei G, Yi S, Yong D, Shaozhuang L, Guangyong Z, Sanyuan H (2018). miR-320 mediates diabetes amelioration after duodenal-jejunal bypass via targeting adipoR1. Surg Obes Relat Dis.

[143] Tomas E, Tsao TS, Saha AK, Murrey HE, Zhang Cc C, Itani SI (2002). Enhanced muscle fat oxidation and glucose transport by ACRP30 globular domain: acetyl-CoA carboxylase inhibition and AMP-activated protein kinase activation. Proc Natl Acad Sci U S A.

[144] Yamauchi T, Kamon J, Minokoshi Y, Ito Y, Waki H, Uchida S (2002). Adiponectin stimulates glucose utilization and fatty-acid oxidation by activating AMP-activated protein kinase. Nat Med.

[145] Kahn BB, Alquier T, Carling D, Hardie DG (2005). AMP-activated protein kinase: ancient energy gauge provides clues to modern understanding of metabolism. Cell Metab.

[146] Donath MY, Ehses JA, Maedler K, Schumann DM, Ellingsgaard H, Eppler E (2005). Mechanisms of beta-cell death in type 2 diabetes. Diabetes.

[147] Mo FF, An T, Zhang ZJ, Liu YF, Liu HX, Pan YY (2017). Jiang Tang Xiao Ke Granule Play an Anti-diabetic Role in Diabetic Mice Pancreatic Tissue by Regulating the mRNAs and MicroRNAs Associated with PI3K-Akt Signaling Pathway. Front Pharmacol.

[148] Yu N, Fang X, Zhao D, Mu Q, Zuo J, Ma Y (2017). Anti-Diabetic Effects of Jiang Tang Xiao Ke Granule via PI3K/Akt Signalling Pathway in Type 2 Diabetes KKAy Mice. PLoS One.

[149] Suksangrat T, Phannasil P, Jitrapakdee S (2019). miRNA Regulation of Glucose and Lipid Metabolism in Relation to Diabetes and Non-alcoholic Fatty Liver Disease. Adv Exp Med Biol.

[150] Ren XP, Wu J, Wang X, Sartor MA, Jones K, Qian J (2009). MicroRNA-320 is involved in the regulation of cardiac ischemia/reperfusion injury by targeting heat-shock protein 20. Circulation.

